# Supplementary material for: ProteinShader: illustrative rendering of macromolecules
Source: BMC Struct Biol. 2009 Mar 30;9:19. doi: 10.1186/1472-6807-9-19 (PMC2672931; doi:10.1186/1472-6807-9-19)
Supplement: Additional file 1 — ProteinShader program without source code. This compressed file contains the complete ProteinShader program including associated libraries, but no source code. A README.txt file gives an overview of the ProteinShader distribution, and the index.html file in the help subdirectory has directions on getting started with the program as well as a set of tutorials. [file 1472-6807-9-19-S1.zip › ProteinShader-beta-0_9_4-binary/help/api/org/proteinshader/structure/class-use/Region.html]

Uses of Class org.proteinshader.structure.Region (ProteinShader API)


|  |  |  |
| --- | --- | --- |
| **Overview** \| **Package** \| **Class** \| **Use** \| **Tree** \| **Deprecated** \| **Index** \| **Help** | |  |
| PREV   NEXT | **FRAMES**    **NO FRAMES**     **All Classes** |  |


---


## **Uses of Class org.proteinshader.structure.Region**

| Packages that use Region | |
| --- | --- |
| **org.proteinshader.structure** | Holds the classes that store information from a Protein Data Bank file: Structure, Model, Chain, AminoAcid, Heterogen, Water, Atom, Bond, Helix, BetaStrand, Loop, *etc*. |
| **org.proteinshader.structure.factory** | Holds the factory classes needed for generating the Segment objects that hold the information needed for rendering ribbon and tube segments. |

Uses of Region in org.proteinshader.structure

| Subclasses of Region in org.proteinshader.structure | |
| --- | --- |
| `class` | `BetaStrand`             This concrete subclass of Region holds information specific to a protein BetaStrand. |
| `class` | `Helix`             This concrete subclass of Region holds information specific to a protein Helix. |
| `class` | `Loop`             This concrete subclass of Region holds information on the general "loop" regions found between well-defined regions of secondary structure (helices and beta-strands). |

Uses of Region in org.proteinshader.structure.factory

| Methods in org.proteinshader.structure.factory with parameters of type Region | |
| --- | --- |
| `LinkedHashMap<String,Segment>` | `SegmentFactory.createSegments(Region region)`             Creates the Segment objects for a Region. |
| `LinkedHashMap<String,Segment>` | `BetaStrandSegmentFactory.createSegments(Region region)`             After using the superclass createSegments() method, the LocalFrames held by some of the Segment objects will be modified so that beta-strands will have a pleated effect rather than a twisted ribbon appearance. |

---


|  |  |  |
| --- | --- | --- |
| **Overview** \| **Package** \| **Class** \| **Use** \| **Tree** \| **Deprecated** \| **Index** \| **Help** | |  |
| PREV   NEXT | **FRAMES**    **NO FRAMES**     **All Classes** |  |


---

# *Copyright © 2007-2008*
